# Astragalosides from Radix Astragali benefits experimental autoimmune encephalomyelitis in C57BL /6 mice at multiple levels

**DOI:** 10.1186/1472-6882-14-313

**Published:** 2014-08-24

**Authors:** Yi-Xin He, Min Du, Hai-Lian Shi, Fei Huang, Hong-Shuai Liu, Hui Wu, Bei-Bei Zhang, Wei Dou, Xiao-Jun Wu, Zheng-Tao Wang

**Affiliations:** Department of Pharmacognosy, China Pharmaceutical University, Nanjing, 210009 China; The Ministry of Education (MOE) Key Laboratory for Standardization of Chinese Medicines, Shanghai Key Laboratory of Complex Prescription, Institute of Chinese Materia Medica, Shanghai University of Traditional Chinese Medicine, Shanghai, 201203 China; Unit of Immune Signaling and Regulation, Institute Pasteur of Shanghai, Chinese Academy of Sciences, Shanghai, 201203 China

**Keywords:** Astragalosides, Experimental autoimmune encephalomyelitis, Multiple sclerosis, Neuroinflammation, Oxidative stress, Apoptosis

## Abstract

**Background:**

Radix Astragali is famous for its beneficial effect on inflammation associated diseases. This study was to assess the efficacy of astragalosides (AST) extracted from Radix Astragali, on the progression of experimental autoimmune encephalomyelitis (EAE), and explore its possible underlying molecular mechanisms.

**Methods:**

EAE was induced by subcutaneous immunization of MOG_35–55_. Infiltration of inflammatory cells was examined by HE staining. ROS level was detected by measuring infiltrated hydroethidine. Leakage of blood brain barrier (BBB) was assessed using Evan’s blue dye extravasation method. Levels of inflammatory cytokines were measured using ELISA kits. Activities of total-SOD, GSH-Px, and iNOS and MDA concentration were measured using biochemical analytic kits. Gene expression was detected using real-time PCR method. Protein expression was assayed using western blotting approach.

**Results:**

AST administration attenuated the progression of EAE in mice remarkably. Further studies manifested that AST treatment inhibited infiltration of inflammatory cells, lessened ROS production and decreased BBB leakage. In peripheral immune-systems, AST up-regulated mRNA expression of transcriptional factors T-bet and Foxp3 but decreased that of RORγt to modulate T cell differentiation. In CNS, AST stopped BBB leakage, reduced ROS production by up-regulation of T-SOD, and reduced neuroinflammation by inhibition of iNOS and other inflammatory cytokines. Moreover, AST inhibited production of p53 and phosphorylation of tau by modulation of the Bcl-2/Bax ratio.

**Conclusions:**

AST orchestrated multiple pathways, including immuno-regulation, anti-oxidative stress, anti-neuroinflammation and anti-neuroapoptosis involved in the MS pathogenesis, to prevent the deterioration of EAE, which paves the way for the application of it in clinical prevention/therapy of MS.

## Background

Multiple sclerosis (MS), a chronic inflammatory demyelinating disease of central nervous system (CNS), generally manifests in an initial relapsing-remitting clinical course that culminates in permanent neurological damage. It is found mostly in young adults in the western world [1, 2]. The main pathological features of the disease include focal CNS inflammation with axonal demyelination and neuronal death [3]. The common clinical strategy for therapy of acute relapses in MS is either by high dose, short-term pulse therapy with glucocorticoid [4] or by immunomodulatory treatments such as interferon beta [5], glatiramer acetate [6], and mitoxantrone [7]. In addition, immunosuppression therapy with drugs such as azathioprine [8], cyclophosphamide [9] and intravenous immune globulin (IVIG) [10] as well as plasmapheresis [11] have also been suggested. Other novel treatments still requiring further clinical trials are estriol [12], statins [13] and natalizumab [14]. Although these drugs can slow down MS progression and ameliorate intensity of relapsed disease, however, long-term therapy with these drugs often gives rise to significant adverse effects including depression, infection, cardiotoxicity, nausea and anemia [15]. Therefore, new therapy with high efficacy but low side-effect is urgently needed for MS treatment.

Experimental autoimmune encephalomyelitis (EAE) is the most widely used animal model to study the pathogenesis and therapeutic interventions of MS. The model can be actively induced by immunizing the animals with different antigenic materials from CNS homogenate, myelin proteins, fusion proteins to small encephalitogenic peptides [16, 17]. Alternatively, autoreactive T cells from immunized animals can be adoptively transferred into naïve animals to induce the disease. Generally, the typical clinical course of EAE exhibits as weight loss, ascending progressive paralysis, and then spontaneous recovery [18]. More importantly, the model mimics the major neuropathological features of MS in histopathology such as inflammation, demyelination, axonal loss and gliosis.

Radix Astragali is the root of *Astragalus membranaceus* Bunge which has been used widely as a key remedy in traditional Chinese medicine for its anti-inflammatory, anti-oxidative, immune-regulatory, and neuro-protective activities [19, 20]. Astragalosides (AST) are the principle bioactive components extracted from roots of Radix Astragali. Recent pharmacological studies have shown that AST benefits the axonal regeneration or growth of both peripheral and central nervous systems. When used at low concentration, AST is salutary in aiding the growth of axons of sciatic nerve [21]. In aged rodents, AST treatment facilitates the recovery of learning and memory impairments [22, 23]. Moreover, a recent report suggests that intravenous infusion of AST in healthy Chinese volunteers is safe and well tolerated [24]. Therefore, AST seems to be an efficient and safe prodrug for the therapy of neurological diseases associated with inflammation.

Astragaloside IV (ASI), one of the single compounds within AST, has been found to attenuate the progression of EAE [25]. However, since ASI within AST was less than 5% according to our study, therefore, whether AST has similar effect has not been known yet. In current study, the effect of AST on C57BL/6 mice induced with EAE by MOG_35–55_ was assessed and compared with that of ASI. Our results demonstrated that AST alleviated the severity of EAE, the efficacy of which was better than that of ASI at the same dose. Thereafter, the underlying mechanisms were discussed from multi-levels involved in the MS pathophsiology, including anti-oxidative stress, anti-inflammation, anti-apoptosis, and immunoregulation. These novel findings provide a new insight into the potential clinical application of AST in therapy or prevention of MS.

## Methods

### Preparation of astragalosides

Decoction pieces of the roots of *Astragalus membranaceus* (Fisch.) Bge were provided by Shanghai Yanghetang Electuary Factory (Shanghai, China) and authenticated by Dr. Hong Xu, Institute of Chinese Materia Medica, Shanghai University of Traditional Chinese Medicine. AST was prepared and purified according to the method mentioned previously [26]. In brief, the 70% ethanol fraction of *A. membranaceus* was sequentially extracted with n-BuOH, purified with macroporous resin, followed by precipitation with diethyl ether:acetone (1:1). The resultant AST was subjected to HPLC analysis to determine the major components as described previously [26]. The percentages of astragaloside I, II and IV in the prepared AST were found to be 76.1, 9.3 and 3.7, respectively.

### EAE induction and AST treatment

The animal experiments were carried out according to the protocols approved by University Animal Care and Use Committee of Shanghai University of Traditional Chinese Medicine. EAE induction was performed in 6-weeks-old female C57BL/6 mice as described previously [25]. Each mouse received 100 μl of complete Freund’s adjuvant emulsified with 300 μg MOG_35–55_ and 400 μg of heat-inactivated *Mycobacterium tuberculosis* H37RA via subcutaneous injection. Pertussis toxin (200 ng/mouse) was administered intraperitoneally (i.p.) immediately and again two days later. Clinical behavior of mice was scored daily in accordance with the criteria described by Peiris et al. [27]. And the day of immunization was considered as EAE day 0.

Daily AST treatments (10, 25, and 50 mg/kg) or ASI (10 mg/kg) were administered i.p. from day 0 to day 14. Methylprednisolone (MPD) treatment served as positive control drug was given i.p. at 20 mg/kg dosage for three consecutive days from day 8 to day 10 post-immunization.

### Histopathology

Mice were anesthetized with 20% urethane and then perfused intracardially with PBS followed by 4% paraformaldehyde. Cross sections of spinal cords at 20 μm thickness were obtained on a Leica 1950 cryostat. Hematoxylin and eosin (HE) staining was conducted to evaluate the extent of infiltration of inflammatory cells.

### Western Blot

Protein samples were obtained by homogenizing brain cortices of mice in CelLytic^™^ MT mammalian tissue lysis reagent (Sigma, C3228) mixed with protease inhibitor cocktail (Sigma, P3840) and phosphatase inhibitor cocktail 2 (Sigma, P5726). The homogenate was centrifuged at 12,000 rpm for 10 min at 4°C. The concentration of the protein was quantified by BCA assay. After being electrophoresed on 12% SDS-PAGE gel, the proteins were transferred onto FluoroTrans^®^ W PVDF membranes (Pall, 20685) via electrophoretic transfer system (Bio-Rad). Then the membranes were blocked with 5% skim milk in PBST for 1 hr followed by incubation with respective primary antibodies at 4°C overnight. After thoroughly washed with PBST, the membranes were further incubated with respective horseradish peroxidase conjugated secondary antibodies. Thereafter, the protein bands were visualized with ECL-prime kit.

### Cytokine quantification

The brain cortices of mice were homogenized in PBS (1:10 w/v) on ice. After being centrifuged at 4000 rpm for 10 min at 4°C, the supernatants were subjected to further ELISA analysis. The concentrations of IFNγ, TNFα, IL6, IL4, and IL17A in brain homogenates were determined using respective ELISA kits referred to the manuals of manufacturer (eBiosciences, San Diego, CA). The cytokine concentrations of respective samples were quantified by standard curves prepared by recombinant cytokines of known concentrations.

### Evan’s blue dye extravasation

To determine the permeability of BBB, the Evan’s blue (EB) dye extravasation method was used as described previously [25]. Briefly, the mice were i.p. injected with 400 μl of 0.8% EB in PBS. Two hours later, the mice were anesthetized with 20% urethane and the brains were dissected and weighed. Hemispheres of brains were homogenized in 1 ml of 50% TCA. After centrifugation at 12,000 rpm for 10 min, the supernatants were collected and subjected to fluorescent intensity detection on a microplate fluorescence reader (excitation :620 nm, emission: 680 nm).

### Reactive oxygen species (ROS) measurements

To assess the ROS level in brain, hydroethidine, the superoxide-sensitive fluorescent probe, was utilized. It is easily oxidized by superoxide into dihydroethidine (DHE), fluorescence of which can be detected by a fluorescence reader. Mice were injected i.p. with 200 μl of 1 mg/ml hydroethidine for 15 min. Thereafter, they were anesthetized with excessive 20% urethane and sacrificed. The brain cortices were dissected and homogenized in CelLytic^TM^ MT mammalian tissue lysis reagent containing protease inhibitor cocktail and phosphatase inhibitor cocktail 2 (1:5, w/v). After centrifugation at 12,000 rpm for 10 min, the supernatants were collected and subjected to fluorescent detection using a Varioskan flash spectral scanning multimode reader (Thermo, excitation :540 nm; emission: 595 nm).

### Biochemical analysis

Samples of brain cortices were prepared as mentioned above in cytokine quantification section. Activities of glutathione peroxidase (GSH-Px), total superoxide dismutase (T-SOD), and inducible nitric oxide synthase (iNOS) and the concentration of malondialdehyde (MDA) in the samples were analyzed with respective kits according to the manuals of manufacturer (Jiancheng Bioengineering Institute, Nanjing, China).

### Quantitative PCR

Hippocampal total RNA of mice was extracted using RNazol according to the manufacturer’s manuals (Takara, Dalian, China). After removal of the trace amounts of DNA contamination with DNase I, total RNA was reverse transcripted into cDNA with kit from Life Technologies (Grand Island, NY, USA). Quantitative PCR was carried out using Taqman SYBR kit (Life Technologies). The concentrations of target genes in the samples were determined by standard curves generated with template plasmids including fragments of the related target genes. At last, they were normalized to that of glyceraldehydes-3-phosphate dehydrogenase (GAPDH) in the same sample. The sequences for all the primers used were described as previously [25].

### Statistical analysis

All data in the graphs were presented as mean±standard error of mean. Statistical comparisons were carried out by one-way analysis of variance (ANOVA) followed by Tukey's post-hoc test using GraphPad Prism 5 software (La Jolla, CA, USA ). Differences were considered as statistically significant when p < 0.05.

## Results

### AST attenuated severity of EAE mice

**Figure 1:**
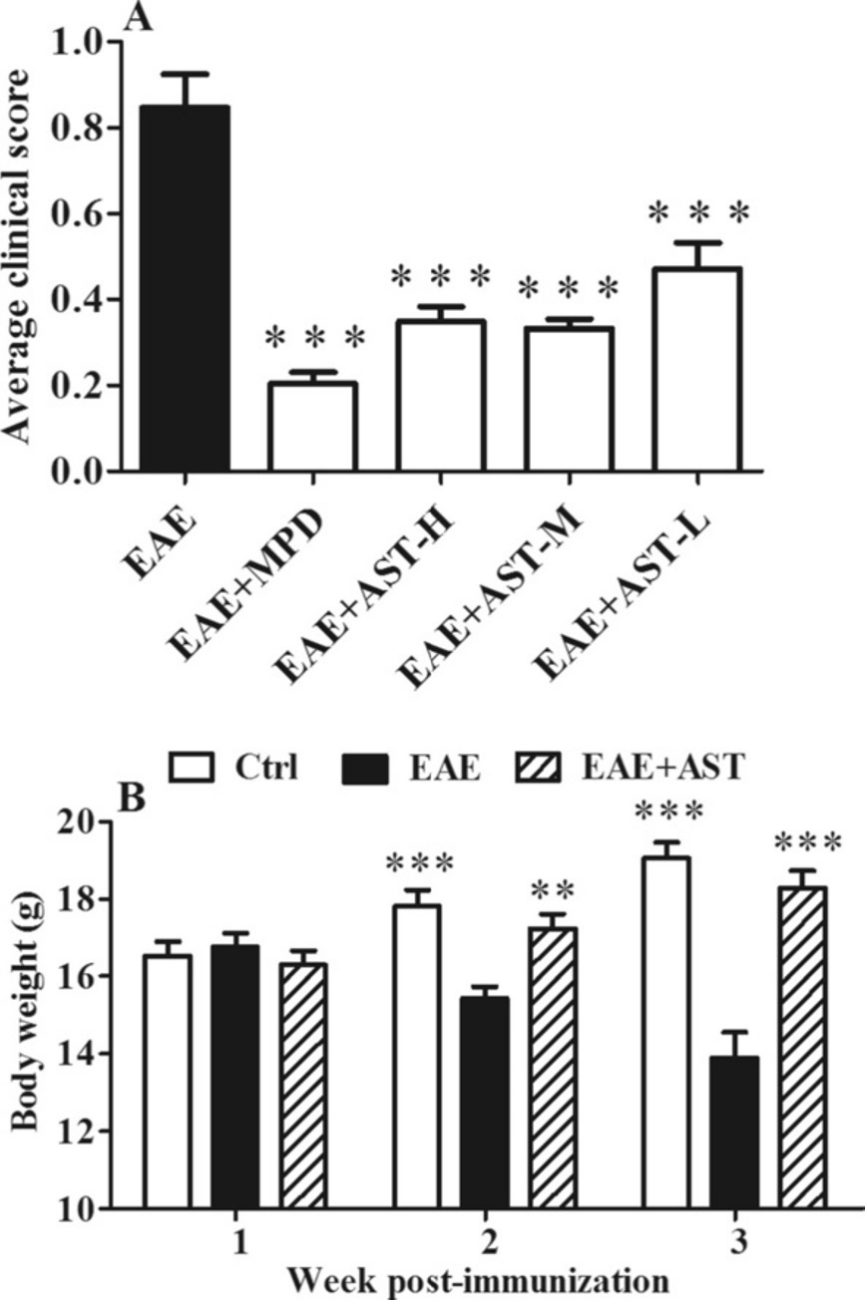
**Effects of AST on EAE progression and body weight in mice. A)** Comparison of mean clinical scores after EAE induction. AST was administered at three different dosages (H: 50 mg/kg/day, M: 25 mg/kg/day, L: 10 mg/kg/day) i.p. from the day before MOG_35–55_ immunization and lasted for 14 days as well as ASI (10 mg/kg/day). Methylprednisolone (MPD) served as positive control drug was given at 50 mg/kg/day i.p. from the day 8 to day 10 post-immunization. **B)** Change of body weight among different group mice. All data are presented as mean±standard error of mean (n = 14). ^**^p < 0.01; ^***^p < 0.001 *vs* EAE group.

Generally, the onset of EAE was started from day 8 after MOG_35–55_ immunization in our experiments. To evaluate the severity of the disease in differently treated mice, the average behavioral scores of mice from day 8 to day 21 post-immunization among groups were compared. As shown in Figure 1A, MPD, the positive control drug, remarkably alleviated the severity of the disease (p < 0.001). AST, when used at doses of 10, 25 and 50 mg/kg, improved the clinical behavioral symptoms of EAE mice significantly (p < 0.001). Meanwhile, AST when used at 25 and 50 mg/kg dosages did not show any difference with MPD regarding the alleviation of EAE severity. However, EAE mice treated with low dose of AST (10 mg/kg) exhibited marked difference with those treated with MPD (p < 0.01).

To examine if the alleviation of EAE by AST was due to ASI it contained, an additional ASI-treated group mice was compared with other AST treated ones. As illustrated in Figure 1A, ASI used at 10 mg/kg also attenuated the deterioration of EAE significantly (p < 0.001), however, its effect seemed to be weaker than that of the middle- and high-dose of AST.

In addition, AST treatment prevented the continuously body weight loss of EAE mice. As illustrated in Figure 1B, compared with normal mice, EAE mice showed prominently body weight loss in two weeks post-immunization and thereafter (p < 0.001). However, AST administration (50 mg/kg) reversed the trend. As shown in Figure 1B, body weight of AST treated EAE mice was gradually increased after MOG immunization.

### AST prevented inflammatory cell infiltration in EAE mice

**Figure 2:**
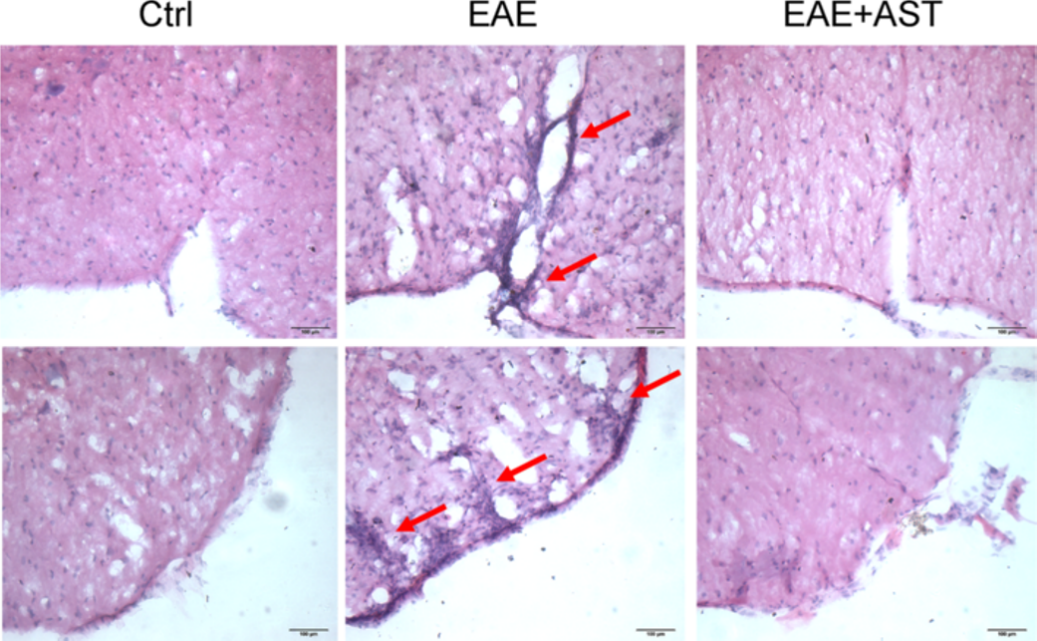
**Effect of AST on inflammatory infiltration of EAE mice.** Pictures denoted HE staining of spinal cords. The dose of AST used was 50 mg/kg/day. Bars in pictures represent 100 μm.

To evaluate the effects of AST (50 mg/kg) on inflammatory cell infiltration in EAE mice, sections of spinal cords of mice were subjected to HE staining. As shown in Figure 2, results from HE staining demonstrated the remarkable infiltration of inflammatory cells in the meanings and parenchymal tissues of spinal cords in EAE mice. By contrast, AST treatment prevented the infiltration of those inflammatory cells.

### AST decreased ROS stress in EAE mice

**Figure 3:**
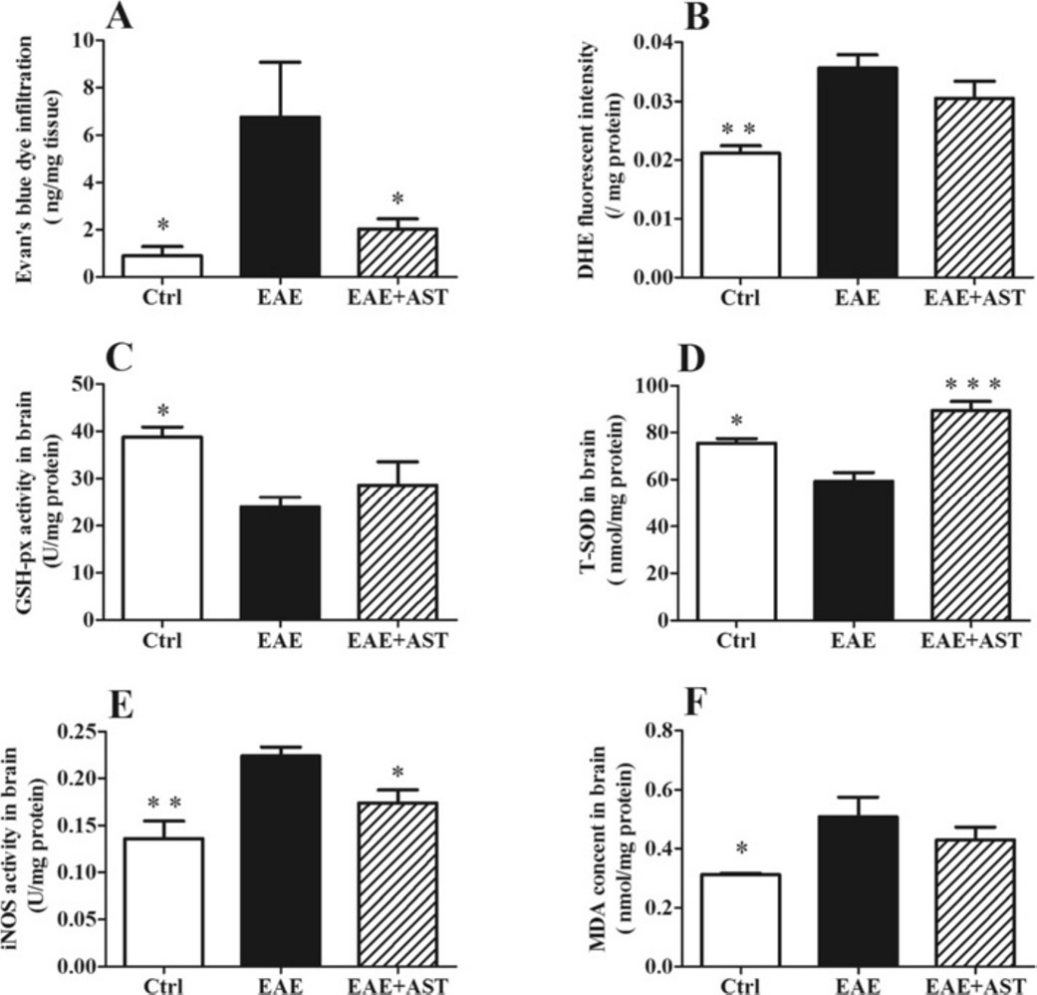
**Effect of AST on permeability of BBB and ROS level. A)** AST decreased permeability of BBB in EAE mice examined by Evan’s blue dye infiltration. **B)** AST reduced ROS release in cortices of EAE mice measured by DHE fluorescent intensity. **C)** AST did not increase GSH-Px activity in cortices of EAE mice. **D)** AST up-regulated T-SOD activity in cortices of EAE mice. **E, F)** AST inhibited elevated iNOS and MDA in cortices of EAE mice. The dose of AST used was 50 mg/kg/day. All data are presented as mean±standard error of mean. n = 5 in each group. ^*^p < 0.05; ^**^p < 0.01; ^***^p < 0.001 *vs* EAE group.

Leakage of BBB and increased oxidative stress are typical phenomena occurred in the pathogenesis of EAE [28]. In our study, the permeability of BBB was assessed by infiltrated Evan’s blue dye. In concordance with previous reports, EAE caused the leakage of BBB even three weeks post-immunization (p < 0.05, Figure 3A). Meanwhile, EAE induced significantly elevated ROS level in CNS of mice. As shown in Figure 3B, fluorescent intensity of infiltrated DHE in EAE mice was increased prominently (p < 0.01), which was accompanied with markedly down-regulated GSH-Px (p < 0.05, Figure 3C) and T-SOD (p < 0.05, Figure 3D) but up-regulated iNOS (p < 0.01, Figure 3E) activity. As a result, MDA, the breakdown product of oxidation of polyunsaturated fatty acids and reliable oxidant marker of oxidative stress-mediated lipid peroxidation [29] generated in brain cortices of EAE mice was increased significantly (p < 0.05, Figure 3F). AST treatment ameliorated the increased BBB leakage of EAE mice (p < 0.05). Although AST did not elevate the activity of GSH-Px, it increased T-SOD activity remarkably (p < 0.01, Figure 3C-D). Moreover, up-regulated iNOS activity was inhibited by AST administration (p < 0.05, Figure 3E). Accordingly, MDA produced in AST treated mice tended to be reduced in spite of no difference in statistics with that in EAE mice received no treatment (Figure 3F).

### AST affected mRNA expression of hippocampal GFAP, CD11b and iNOS

**Figure 4:**
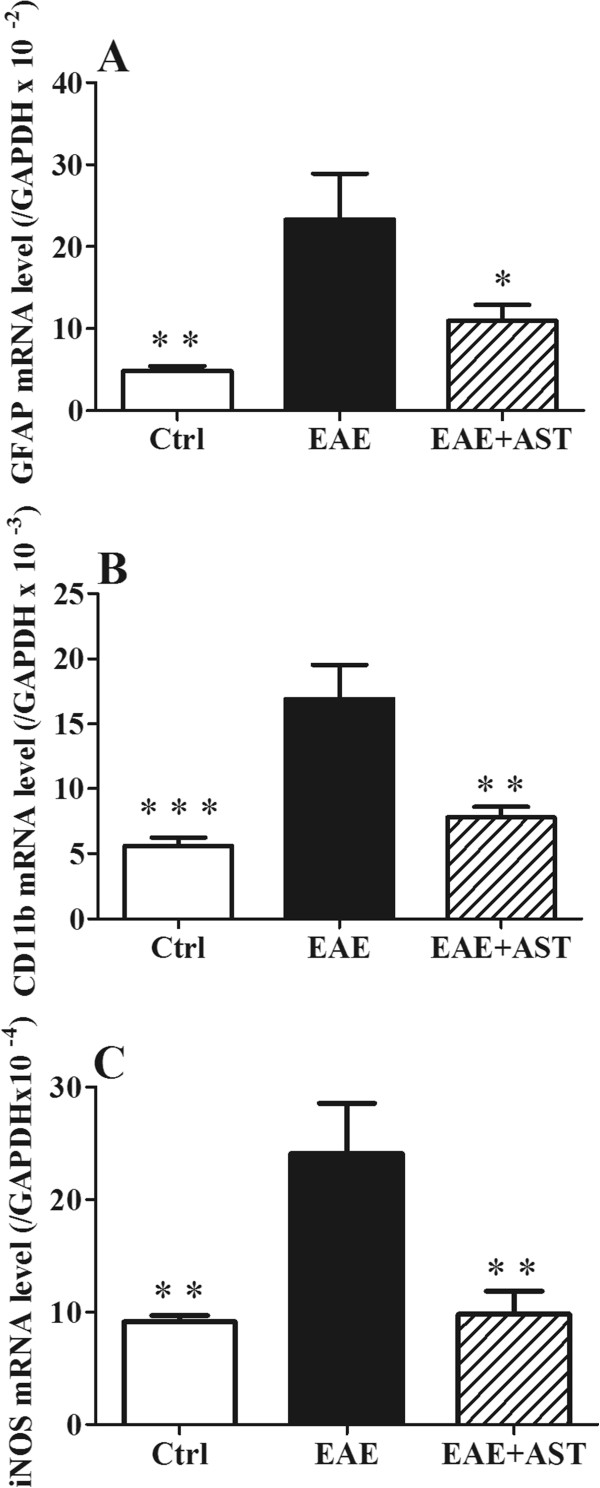
**Effect of AST on hippocampal mRNA expression of GFAP, CD11b and iNOS in EAE mice. A)** AST inhibited GFAP mRNA expression. **B)** AST reduced CD11b mRNA expression. **C)** AST prevented iNOS mRNA expression. All data are presented as mean±standard error of mean. The dose of AST used was 50 mg/kg/day. n = 5 in each group. ^*^p < 0.05; ^**^p < 0.01 *vs* EAE group.

GFAP and CD11b are astroglial and microglial markers in CNS, respectively. EAE caused significantly enhanced mRNA expression of both GFAP (p < 0.01, Figure 4A) and CD11b (p < 0.001, Figure 4B) in hippocampus. Meanwhile, mRNA expression of iNOS was also up-regulated (p < 0.01, Figure 4C). When AST was administered, the GFAP, CD11b, and iNOS mRNA expression of EAE mice were all down-regulated significantly (p < 0.05, p < 0.01, and p < 0.01, respectively, Figure 4A-C).

### AST affected mRNA expression of splenic RORγt, T-bet, and Foxp3

**Figure 5:**
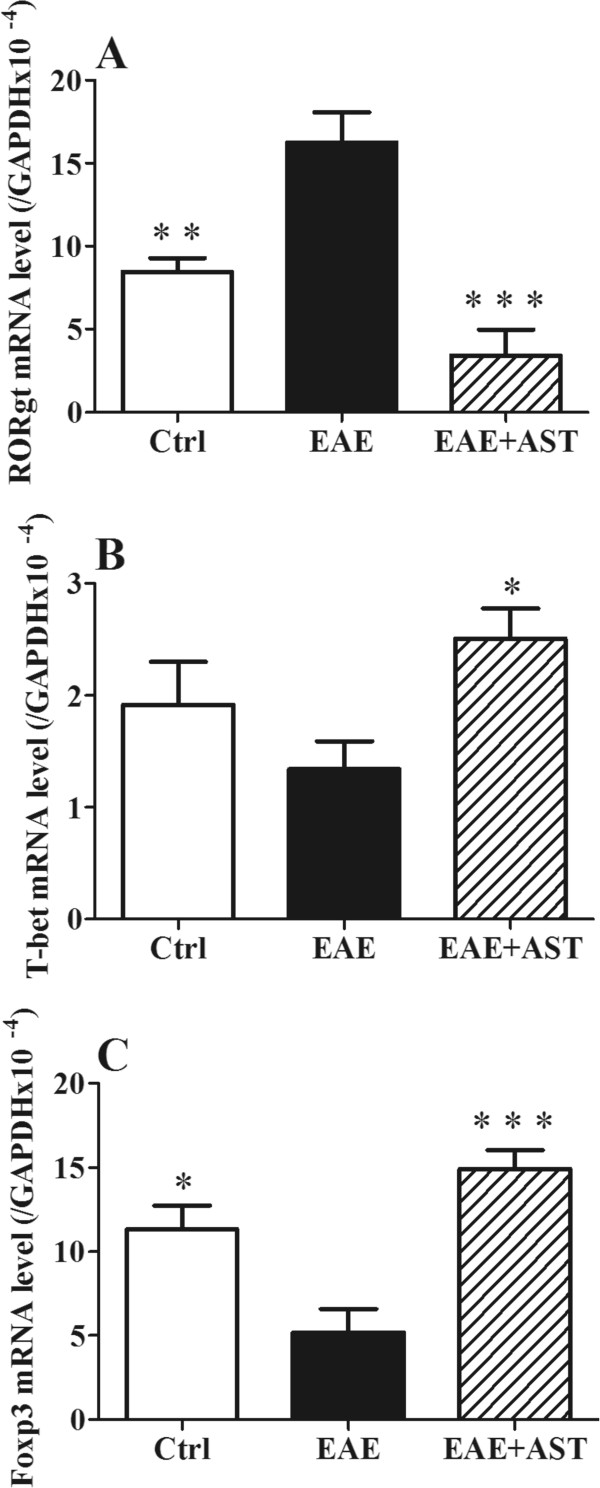
**Effect of AST on splenic mRNA expression of RORγt, T-bet, and Foxp3 of EAE mice. A)** AST inhibited RORγt mRNA expression. **B)** AST increased T-bet mRNA expression. **C)** AST elevated Foxp3 mRNA expression. All data are presented as mean±standard error of the mean. The dose of AST used was 50 mg/kg/day. n = 5 in each group. ^*^p < 0.05; ^**^p < 0.01; ^***^p < 0.001 *vs* EAE group.

RORγt, T-bet and Foxp3 are transcription factors associated with the differentiation of CD4+ T cells into Th1, Th17 and Treg effector cells. In our study, the significant increase of RORγt mRNA expression in spleens of EAE mice (p < 0.01, Figure 5A), was abrogated by AST administration (50 mg/kg). Although splenic mRNA expression of T-bet did not change in EAE mice, it was prominently elevated by AST treatment (p < 0.05, Figure 5B). Meanwhile, splenic Foxp3 mRNA expression was down-regulated in EAE mice, which was compensated remarkably by AST treatment (p < 0.01, Figure 5C).

### AST regulated cytokine profile of EAE mice

**Figure 6:**
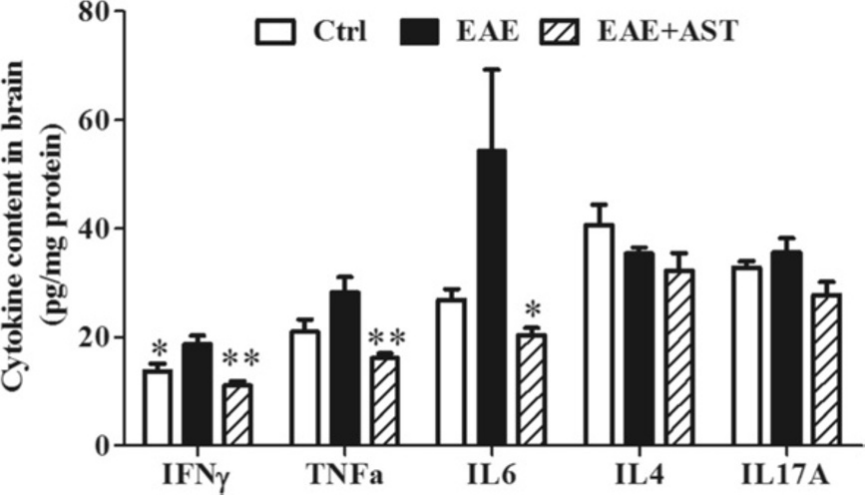
**Effect of AST on cytokine profile in cortices of EAE mice.** All data are presented as mean±standard error of mean. The dose of AST used was 50 mg/kg/day. n = 5 in each group. ^*^p <0.05; ^**^p < 0.01 *vs* EAE group.

To assess the effect of AST on cytokine profile of EAE mice, brain cortices of mice three weeks post-immunization were homogenized and the supernatants after centrifugation were subjected to respective cytokine analysis using different ELISA kits. As shown in Figure 6, concentrations of most cytokines examined including TNFα, IL6, IL4 and IL17A in EAE mice did not differ from that in control mice except IFNγ (p < 0.05). AST treatment did not affect IL4 and IL17A concentrations in brain cortices of EAE mice. However, it decreased IFNγ (p < 0.01), TNFα (p < 0.01) and IL6 (p < 0.05) levels.

### AST modulated expression of apoptotic proteins in CNS

**Figure 7:**
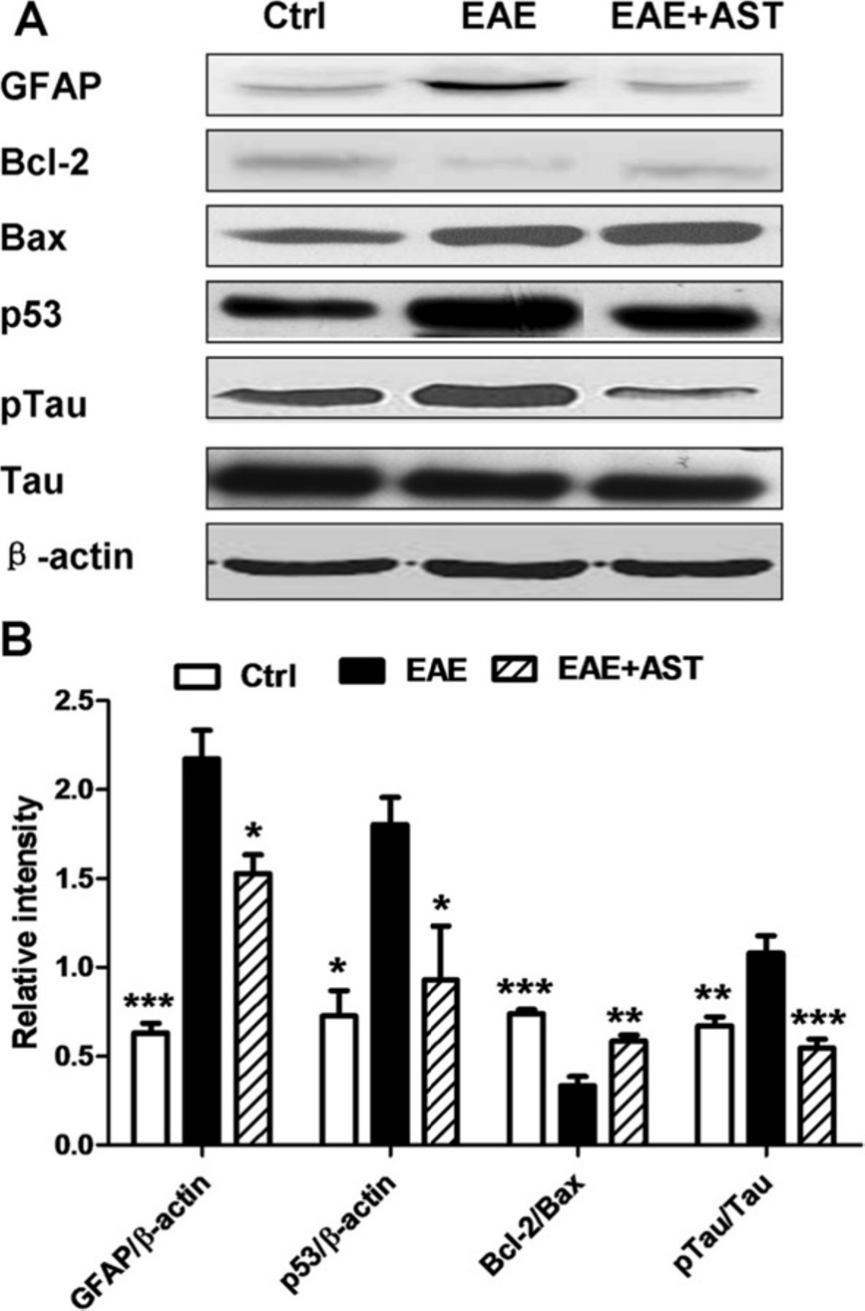
**Effect of AST on expression of apoptotic proteins in cortices of EAE mice. A)** Typical bands of GFAP, Bcl-2, Bax, p53, p-tau and tau in cortices of EAE mice treated with AST detected by western blotting. **B)** Gray intensity analysis of GFAP, Bcl-2, p53 and p-tau. All data are presented as mean±standard error of mean. The dose of AST used was 50 mg/kg/day. n = 5 for each group. ^*^p < 0.05; ^**^p < 0.01; ^***^p < 0.001 *vs* EAE group.

Neuronal injury caused by infiltrated inflammatory T cells is a common feature of EAE. To evaluate the effect of AST treatment on neuronal damage, expression of proteins associated with neuronal apoptosis in cortices of mice were analyzed. Consistent with the mRNA expression pattern, GFAP protein expression in EAE mice was elevated markedly (p < 0.001, Figure 7). The pro-apoptotic protein, p53, was also up-regulated (p < 0.05). Accordingly, the phosphorylated tau protein that indicates the extent of neuronal injury was increased prominently (p < 0.01). Expression of Bax, one of the members of Bcl-2 family, was also enhanced. On the contrary, Bcl-2, the anti-apoptotic protein, was inhibited in EAE mice. AST treatment (50 mg/kg) prevented further neuroinflammation and apoptosis of neuronal cells. Compared with EAE mice without any treatment, protein expression of GFAP and p53 in AST treated EAE mice were inhibited remarkably (p < 0.05). Although Bax expression was not inhibited by AST treatment, the ratio of Bcl-2 to Bax was significantly increased (p < 0.01). Therefore, the phosphorylation of tau in EAE mice was prevented markedly (p < 0.001).

## Discussion

In present study, AST treatment attenuated the progression of EAE mice significantly. Further studies demonstrated that multiple pathways mediated the preventive effect of AST. Increased inflammatory cell infiltration, BBB leakage, and ROS level in CNS were the significant features of EAE mice. AST administration counteracted the harmful effect by modulating the differentiation of T cells, increase of anti-oxidant enzymes, and inhibition of neuroinflammation. In addition, in CNS it mitigated the neuronal apoptosis during EAE by modulating the ratio of Bcl-2 to Bax, therefore, reduced the phosphorylation of tau and thus neuronal damages in CNS.

AST was the total astragalosides extracted from *A. membranaceus* that mainly composed of astragaloside I, II and IV according to our analysis. And astragaloside I was the major component within AST (>76%). However, since AST also contained 3.7% astragaloside IV (ASI), the compound that had been reported by our group to alleviate the progression of EAE [25], the effect of AST on EAE might be due to ASI. To examine the possibility, we added one group of EAE mice treated with ASI (10 mg/kg). According to our result, not surprisingly, ASI prevented the aggravation of EAE as we reported previously. However, the effect of ASI seemed to be weaker than AST (25 mg/kg and 50 mg/kg), which contained less than 2 mg/kg of ASI. These findings indicated that other components besides ASI in AST might also contribute to the alleviative effect, or that many ingredients of AST may have a synergistic alleviative effect, which should be investigated in further studies.

T helper cell subsets, Th1, Th17 and Tregs (regulatory T-cells), play essential regulatory roles in MS or EAE pathogenesis. EAE was easily induced in mice adoptively transferred with MOG-specific Th1 cells [30]. Recently, Th17 cells are thought to have a pivotal role in the pathogenesis of EAE. In vitro differentiated Th17 cells, when adoptively transferred to mice, will form specific immune synapse-like contacts with neurons and induce the death of latter [31]. By contrast, the induction or transplant of Tregs leads to reduction of disease severity in EAE [32]. Transcriptional factors, T-bet, RORγt and Foxp3, contribute to the differentiation of CD4 naïve T cells into respective subsets. In the presence of IL12 and expression of T-bet, Th1 cells can be generated from naïve T cells; when transforming growth factor (TGF)-β plus IL 6 are present with the expression of RORγt, Th17 cells will be generated [33]. Foxp3, as a master regulator, is important for the development and function of Tregs [34]. In our experiments, all of the three transcriptional factors were modulated by AST administration at mRNA levels (Figure 5), which indicated the role of AST in control of the differentiation of naïve T cells. Therefore, there were less inflammatory T cells infiltrated into CNS of EAE mice after treatment with AST (Figure 2).

Opening or breakdown of BBB has been known to be involved in the pathogenesis of MS or EAE [35, 36]. Meanwhile, ROS leading to oxidative stress that is produced primarily by infiltrated macrophages have been suggested as mediators of demyelination and axonal damages in MS and EAE [37]. In turn, increased ROS can cause enhanced permeability of the BBB [38]. Consistent with the reports, our study showed that EAE progression caused marked leakage of BBB and elevation of ROS in CNS (Figure 3). As a result, both of MDA and phosphorylated tau, the indicators to evaluate the extent of neuronal damage, were accumulated significantly (Figures 3 and 7). AST administration prevented the leakage of BBB and reduced ROS level in CNS. Both of GSH-Px and T-SOD function as part of anti-oxidant defense system. Our studies disclosed that AST mainly increased the activity of T-SOD but not GSH-Px, therefore, enhanced the ROS scavenging capacity of CNS.

Gliosis, i.e. astrocytosis and microgliosis, within and around the inflammatory demyelinating lesions is one of the prominent features of both MS and EAE [39, 40]. Reactive astrocytes on the one hand trigger innate proinflammatory response after CNS injury and on the other hand form scar-like perivascular barriers to restrict the influx of leukocytes into CNS parenchyma [41]. As the innate immune cells in CNS, microglia are necessary for normal brain function to help host defense by eliminating invading pathogens, removing deleterious debris, accelerating tissue repair and facilitating tissue homeostasis [42]. However, uncontrolled and sustained activation of microglia will generate excessive detrimental substances such as nitric oxide, free radicals and proinflammatory cytokines that finally result in neuronal destruction [43]. In our experiments, significantly increased mRNA levels of both GFAP and CD11b were found in CNS of EAE mice accompanied with elevated iNOS activity, which indicated the occurrence of neuroinflammation. After AST treatment, the activation of astrocytes and microglia cells was inhibited as the mRNA levels of both GFAP and CD11b were reduced remarkably (Figure 4).

p53 plays a suppressive role in the inflammatory response by regulation the cytokine profile as well as the destiny of infiltrated cells [43]. Abnormalities in p53 at the lesion site may influence the severity or chronicity of MS [44]. Deficiency of p53 increases the severity of EAE possibly by elongating the survival of inflammatory cells in the CNS, therefore, enhancing the production of proinflammatory cytokines such as IL-6, TNF-α and IFN-γ. In current study, p53 level was elevated markedly in CNS of EAE mice but accompanied with increased IFN-γ, which might reflect a self-defense mechanism in anti-inflammation. AST administration prohibited further neuroinflammation as the production of IFN-γ, TNF-α and IL 6 was inhibited significantly. Meanwhile, the level of p53 was resumed to normal condition.

Both Bcl-2 and Bax belong to Bcl-2 family but play different role in control of apoptotic cascade of cells. The balance of them determines the fate of cells toward survival or death, therefore, the ratio of Bcl-2 to Bax is a better determinant to evaluate the apoptotic tendency of cells [44, 45]. In our study, Bax was found to be elevated significantly in CNS of EAE mice (Figure 7). AST treatment did not mitigate the level of Bax but recovered or prevented the degradation of Bcl-2 relatively, therefore, increased the ratio of Bcl-2 to Bax. As a result, the phosphorylated tau protein in CNS was decreased remarkably. Pathological hyperphosphorylation and aggregation of microtubule-associated protein tau is a common character of many neurodegenerative diseases with axonal degeneration including MS [45].

## Conclusion

In summary, AST administration alleviated the progression of EAE by interfering multiple aspects involved in the MS pathogenesis, including immuno-regulation, anti-oxidative stress, anti-neuroinflammation and anti-neuroapoptosis. This study paves the elementary way for the potential application of AST in clinical intervention of MS.

## Abbreviations

AST: Astragalosides
EAE: Experimental autoimmune encephalomyelitis
BBB: Blood brain barrier
MS: Multiple sclerosis
CNS: Ccentral nervous system
IVIG: Iintravenous immune globulin
i.p.: Intraperitoneally
MPD: Methylprednisolone
HE: Hematoxylin and eosin
EB: Evan’s blue
ROS: Reactive oxygen species
DHE: dihydroethidine
GSH-Px: glutathione peroxidase
T-SOD: Total superoxide dismutase
iNOS: inducible nitric oxide synthase
MDA: Malondialdehyde
GAPDH: glyceraldehydes-3-phosphate dehydrogenase
ANOVA: One-way analysis of variance
ASI: Astragaloside IV.
